# P-9. Direct Identification of Gram-Positive Bacteria Culture: Analytical Performance of a Multi-pathogen Molecular Assay

**DOI:** 10.1093/ofid/ofaf695.240

**Published:** 2026-01-11

**Authors:** Christine Pepich, Samantha Coons, Seema Shaikh, Brian Bernier, Amanda Seyfert, Janet Farhang, Sundaresh Brahmasandra

**Affiliations:** Diasorin, Northbrook, Illinois; Diasorin, Northbrook, Illinois; Diasorin, Northbrook, Illinois; Diasorin, Northbrook, Illinois; Diasorin, Northbrook, Illinois; Diasorin, Northbrook, Illinois; Diasorin, Northbrook, Illinois

## Abstract

**Background:**

Bloodstream infection (BSI) is a serious and life-threatening condition requiring timely diagnosis and treatment. This study evaluated a multi-target molecular assay under development for rapid and accurate identification of gram-positive (GP) bacteria directly from blood culture specimens. Here, we report progress in developing a multiplexed gram-positive blood culture assay in development to identify 13 pathogens and 3 resistance markers in approximately 2 hours.

**Methods:**

The assay was performed directly on blood culture positive specimens determined to contain a GP organism. A 300 µL aliquot was processed in a single-use cartridge, utilizing a sensitive hybridization-based assay for rapid, direct detection without the need for target amplification. This fully automated system performs nucleic acid extraction followed by hybridization to a microarray with capture probes specifically designed to detect the clinically relevant gram-positive bacteria and resistance genes via gold nanoparticle chemistry with silver-enhanced signal amplification.

**Results:**

The prototype assay demonstrated 100% positivity for GP targets and 0% for negative blood cultures in a growth and detection study from ring positive bottles and bottles grown up to 8 hours past ring positivity. Over 1,500 samples were tested as part of a site-to-site reproducibility study including 16 blood culture samples comprised of 7 on-panel organisms, one off-panel organism, and one negative across 3 sites resulting in 99.7% assay reproducibility. It accurately detected all targets, including resistance markers, across 13 media types and demonstrated 100% inclusivity for 183 on-panel strains, including resistance markers. The prototype assay maintained sensitivity even with low concentrations of on-panel GP organisms in the presence of high levels of other co-infecting GP or gram-negative bacteria. Clinical validation is underway with over 600 prospective samples, supporting the ongoing assessment of the assay's real-world performance.

**Conclusion:**

This rapid, highly sensitive prototype assay accurately detects GP pathogens and resistance markers, supporting timely BSI management directly from positive blood culture samples and without target amplification.

**Disclosures:**

Christine Pepich, BS, Diasorin: Employee Samantha Coons, BS, DiaSorin: Employee Seema Shaikh, MS, Diasorin: Employee Brian Bernier, PhD, Diasorin: Employee Amanda Seyfert, MS, Diasorin: Employee Janet Farhang, PhD, Diasorin: Employee|Diasorin: Employee Sundaresh Brahmasandra, PhD, Diasorin: Employee

